# An Immunochip-based interrogation of scleroderma susceptibility variants identifies a novel association at *DNASE1L3*

**DOI:** 10.1186/s13075-014-0438-8

**Published:** 2014-10-21

**Authors:** Jane Zochling, Felicity Newell, Jac C Charlesworth, Paul Leo, Jim Stankovich, Adrian Cortes, Yuan Zhou, Wendy Stevens, Joanne Sahhar, Janet Roddy, Peter Nash, Kathleen Tymms, Maureen Rischmueller, Sue Lester, Susanna Proudman, Matthew A Brown

**Affiliations:** Menzies Research Institute Tasmania, University of Tasmania, 17 Liverpool Street, Hobart, TAS 7000 Australia; University of Queensland Diamantina Institute, Translational Research Institute, Princess Alexandra Hospital, 37 Kent Street, Woolloongabba, Brisbane, QLD 4102 Australia; Rheumatology Department, St Vincent’s Hospital, 41 Victoria Parade, Fitzroy, Melbourne, VIC 3065 Australia; Department of Rheumatology, Monash Medical Centre, 246 Clayton Road, Clayton, Melbourne, VIC 3168 Australia; Department of Rheumatology, Royal Perth Hospital, 197 Wellington Street, Perth, WA 6000 Australia; Research Unit, Sunshine Coast Rheumatology, Denna Street, Maroochydore, QLD 4558 Australia; Canberra Rheumatology, 40 Marcus Clarke Street, Canberra, ACT 2601 Australia; Department of Rheumatology, The Queen Elizabeth Hospital, 28 Woodville Road, Woodville, SA 5011 Australia; Rheumatology Unit, Royal Adelaide Hospital, North Terrace, Adelaide, SA 5000 Australia

## Abstract

**Introduction:**

The aim of the study was to interrogate the genetic architecture and autoimmune pleiotropy of scleroderma susceptibility in the Australian population.

**Methods:**

We genotyped individuals from a well-characterized cohort of Australian scleroderma patients with the Immunochip, a custom array enriched for single nucleotide polymorphisms (SNPs) at immune loci. Controls were taken from the 1958 British Birth Cohort. After data cleaning and adjusting for population stratification the final dataset consisted of 486 cases, 4,458 controls and 146,525 SNPs. Association analyses were conducted using logistic regression in PLINK. A replication study was performed using 833 cases and 1,938 controls.

**Results:**

A total of eight loci with suggestive association (*P* <10^-4.5^) were identified, of which five showed significant association in the replication cohort (*HLA-DRB1, DNASE1L3, STAT4, TNP03-IRF5* and *VCAM1*). The most notable findings were at the *DNASE1L3* locus, previously associated with systemic lupus erythematosus, and *VCAM1*, a locus not previously associated with human disease. This study identified a likely functional variant influencing scleroderma susceptibility at the *DNASE1L3* locus; a missense polymorphism rs35677470 in *DNASE1L3,* with an odds ratio of 2.35 (*P* = 2.3 × 10^−10^) in anti-centromere antibody (ACA) positive cases.

**Conclusions:**

This pilot study has confirmed previously reported scleroderma associations, revealed further genetic overlap between scleroderma and systemic lupus erythematosus, and identified a putative novel scleroderma susceptibility locus.

**Electronic supplementary material:**

The online version of this article (doi:10.1186/s13075-014-0438-8) contains supplementary material, which is available to authorized users.

## Introduction

Systemic sclerosis (SSc) or scleroderma is a multisystem, autoimmune disorder characterised by progressive vascular, inflammatory and fibrotic dysfunction. Skin and visceral complications of cardiac, pulmonary, gastro-intestinal, muscle and renal disease can have devastating effects on quality of life and life expectancy [1].

Scleroderma has a well-established genetic component [2-4]. Most of the identified SSc susceptibility loci overlap with those of other autoimmune diseases, in particular the rheumatic disorders such as rheumatoid arthritis and systemic lupus erythematosus (SLE) [5]. For example, Carmona *et al.* recently confirmed an association between SSc and the SLE risk haplotype at the *IRF5* locus [6] and Martin *et al.* recently performed a pan-meta-analysis of SSc and SLE to look at the susceptibility overlap between the two diseases [7]. Using 6,835 cases and 14,274 controls they identified a novel pleiotropic locus at *KIAA0319L* on chromosome 1 and identified two SLE loci (near *PXK* and *JAZF1*) that also contribute to SSc [7].

To identify further susceptibility loci and to explore the genetic overlap with other antibody-mediated immune diseases, we undertook an SSc association study using the Immunochip, a custom array including SNPs of interest in a wide variety of autoimmune disorders [8]. A replication study was then performed using previously published case data from a genome-wide association study (GWAS) of scleroderma [9], and control data from a GWAS of bone density variation [10].

## Methods

### Samples

We selected 532 cases for genotyping from the Australian Scleroderma Cohort Study (ASCS) [11]; a prospective study of risk factors for clinically important outcomes in SSc. They fulfilled either the American College of Rheumatology (ACR) criteria for classification of SSc [12] or the Medsger criteria for limited SSc (to enable broad representation of the disease spectrum) [13]. The study was approved by the Human Research Ethics Committee (Tasmanian) Network and human research ethics committees of St. Vincent’s Hospital and Monash Medical Centre, Melbourne, VIC; Sunshine Coast Rheumatology, Maroochydore, QLD; Royal Adelaide Hospital, Adelaide, SA; St George Hospital, Sydney, NSW; Royal Perth Hospital, Perth, WA; and Prince Charles Hospital, Brisbane, QLD. All patients gave written, informed consent.

### Genotyping

Cases were genotyped with the Immunochip, an Illumina Infinium SNP microarray (Illumina Inc., San Diego, CA, USA) [8], at the University of Queensland Diamantina Institute, Brisbane, QLD, Australia. The Immunochip contains 195,806 common and rare SNPs of interest in a wide variety of autoimmune disorders. Control genotypes were obtained from 4,537 samples from the 1958 British Birth Cohort [14]. Genotypes were called using the Illumina GenTrain clustering algorithm. Cases and controls were clustered separately.

For replication purposes, genotypes from 833 SSc cases were obtained from dbGAP (dbGaP Study Accession: phs000357.v1.p1) [9,15]. These samples were genotyped with the Illumina Human610-Quad v1.0 BeadChip. Control genotypes were obtained from 1,938 subjects of white British ancestry genotyped as part of the Anglo-Australasian Osteoporosis Genetics Consortium program using either Illumina Infinium II HumHap300 or 370CNV chips [10].

### Statistical analyses

Genotype data were analysed with PLINK [16] and R [17]. There were no duplicate or closely related cases. Case (n = 2) and control (n = 3) samples with call rates less than 90% were excluded. SNPs were excluded based on Hardy-Weinberg disequilibrium (*P* <10^−6^), call rates less than 90%, fewer than two occurrences of the minor allele, and significantly different rates of missingness (*P* <10^−4^) between cases and controls. Eigenstrat [18] was run on a pruned SNP set with default settings to exclude population ancestry outliers and ensure cases and controls were ethnically matched. Subjects lying more than six standard deviations from the mean of any principal component were excluded (Immunochip set 44 cases and 76 controls excluded; replication set 129 cases and 31 controls excluded).

Four-digit classical MHC allele dosages at *HLA-A, HLA-B, HLA-C, HLA-DRB1, HLA-DQA1* and *HLA-DQB1* were imputed using HLA*IMP [19,20] in the Immunochip dataset, and tested for association*.*

Logistic regression was used for all association analyses using principal components derived from the Eigenstrat analysis as covariates to control for population stratification. A single principal component was used for the Immunochip analysis and two principal components for the replication dataset; including further principal components for either set did not reduce the genomic inflation factor further. A negative control set of 2,805 SNPs outside the major histocompatibility complex (MHC), associated with reading and learning, schizophrenia and psychosis [19], was used to estimate the genomic inflation factor and calculate adjusted *P* values. Genotype intensity cluster plots were manually examined for all suggestive associations with unadjusted *P* values less than 10^–4.5^, a threshold used in previous GWAS [21]. To test for secondary association signals at each locus, genotypes at the most significant variant were added to the logistic regression model as a covariate, and all other variants at the locus were tested. To correct secondary analyses for multiple testing, we estimated the effective number of independent tests at loci using the eigenvalues of the matrix of correlations between SNPs [22], as implemented in SNPSpD [23].

For variants associated with SSc at the nominally suggestive threshold (*P* <10^-4.5^), we also tested for differences in allele frequencies between patients with different disease subtypes and MHC genotypes.

For replication, cases and controls genotypes were imputed as implemented in IMPUTE2 [24] with the use of the merged 1000 Genomes and UK10K reference dataset. All SNPs we were attempting to replicate had an info score of >0.7. Meta-analysis was performed using METAL weighted by inverse variance [25]. Power was calculated using the Genetic Power Calculator [26].

## Results

After sample and SNP exclusions, in the Immunochip dataset genotypes were analysed at 145,921 autosomal SNPs in 486 cases and 4,458 controls, with a genomic inflation factor of 1.02 (Q-Q plot, Figure S1 in Additional file 1). Eighty-six percent of the cases were female, mean age was 60 years and mean disease duration 14 years. Twenty-five percent of cases had diffuse disease, 72% limited pattern, and 3% were intermediate; 43% were anti-centromere antibody (ACA) positive and 15% anti-Scl-70 antibody positive. Considering the replication set, 700 scleroderma cases and 1,899 controls remained after quality control. Of the replication cases, 36% had diffuse disease, 64% limited pattern; 32% were ACA positive. The genomic inflation factor for the replication set was 1.045. The Immunochip study has 80% power to detect associations at *P* <10^-4.5^ for a variant with minor allele frequency (MAF) of 0.3 with D’ = 0.8 with a SNP with heterozygote odds ratio (OR) of 1.65.

**Figure 1:**
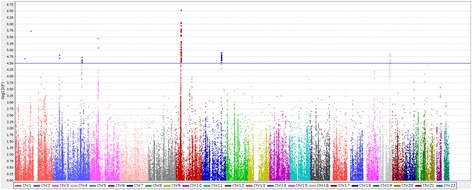
**Manhattan Plot for association study finding from Immunochip genotyped cases and controls.**

We detected suggestive associations in the Immunochip data (uncorrected *P* <10^–4.5^) at eight loci (Table 1), five of which showed association in the replication cohort (*HLA*-*DRB1*, *DNASE1L3, STAT4, TNPO3-IRF5,* and *VCAM1*) (Table 1, Manhattan plot Figure 1)*.* There was also evidence of association at the previously reported genome-wide significant *CD247* SNP [9] (rs2056626, OR = 0.76, *P* = 1.1 × 10^−4^), but no evidence of association at the genome-wide significant *TNIP1* locus [27] (rs2233287, *P* = 0.94). The overlap between previously reported genome-wide significant SSc loci (outside the MHC) and our data, at an unadjusted *P* <0.01, is shown in Table 2. Not all previously associated SSc loci could be investigated owing to the limitations of markers available on the array.

**Table 1:** **SNPs associated with SSc (** ***P*** **<10** ^**–4.5**^ **) in an analysis of Immunochip genotypes for 486 cases and 4,458 controls, replication cohort of 700 SSc cases (220 ACA positive) and 1,889 controls, and combined**

**Chr**	**Location range (base pairs, hg18)**	**Best SNP**	**MAF cases**	**MAF controls**	**Immunochip OR**	**Immunochip** ***P*** **value**	**Replication OR**	**Replication** ***P*** **value**	**Combined analysis**	**Gene annotation** ^**#**^
**6**	32,039,116-32,888,448	rs2857130	0.293	0.389	0.68	2.8 × 10^−7^	0.81	0.003	1.3 × 10^−8^	Intergenic (HLA region)
**1**	101,009,225	Novel SNP	0.024	0.008	3.31	1.8 × 10^−6^	2.49	0.031	1.9 × 10^−7^	Intergenic (*VCAM1*)
**3**	58,158,676-58,289,303	rs35677470	0.122	0.083	1.63	3.4 × 10^−6^	1.27	0.027	1.2 × 10^−6^	*DNASE1L3* (coding)
**3**	58,158,676-58,289,303	rs35677470	0.174	0.083	2.36	2.3 × 10^−10^	1.74	3.0 × 10^−4^	8.71 × 10^−13^	*DNASE1L3* (coding)^^^
**7**	128,372,852-128,499,110	rs34381587	0.158	0.113	1.53	1.2 × 10^−5^	1.38	8.7 × 10^−4^	5.2 × 10^−8^	*IRF5/TNPO3* (intron)
**16**	73,863,956-74,046,823	rs11149824	0.469	0.391	1.35	1.4 × 10^−5^	1.054	0.442	3.2 × 10^−4^	*CFDP1* (intron)
**2**	43,775,459-43,784,213	rs13403030	0.384	0.318	1.36	1.5 × 10^−5^	0.92	0.242	0.027	*PLEKHH2* (intron)
**2**	191,608,694-191,641,499	rs13426947	0.253	0.191	1.42	1.8 × 10^−5^	1.25	5.4 × 10^−3^	6.1 × 10^−7^	*STAT4* (intron)
**1**	61,883,642	rs2886326	0.249	0.195	1.41	2.0 × 10^−5^	0.96	0.606	7.4 × 10^−3^	Intergenic (*TM2D1*)

^#^Gene annotation is based on the location of the most significant SNP; ^^^ACA-positive cases only. SNP, single nucleotide polymorphism; SSc, systemic sclerosis; ACA, anti-centromere antibody; Chr, chromosome; MAF, minor allele frequency; OR, odds ratio.

**Table 2:** **Association finding for previously reported loci achieving** ***P*** **<0.01 in Immunochip, with findings in replication and overall datasets**

**SNP**	**Chr**	**Location (hg18; bp)**	**Locus**	**MAF cases**	**MAF controls**	**OR**	**Unadjusted** ***P*** **value**	**Replication OR**	**Replication** ***P*** **value**	**Combined analysis**	**References**
rs2056626	1	165,687,049	*CD247*	0.359	0.428	0.76	1.1 × 10^−4^	0.84	0.007	3.9 × 10^−6^	[6,7,11]
rs3821236	2	191,611,003	*STAT4*	0.255	0.194	1.41	2.6 × 10^−5^	1.24	0.008	1.2 × 10^−6^	[6,7]
rs10168266	2	191,644,049	*STAT4*	0.249	0.190	1.40	4.1 × 10^−5^	1.30	0.001	1.9 × 10^−7^	[11]
rs7574865	2	191,672,878	*STAT4*	0.286	0.225	1.35	1.2 × 10^−4^	1.15	0.066	5.7 × 10^−5^	[7]
rs2176082	3	58,306,226	*PXK*	0.343	0.296	1.31	2.6 × 10^−4^	1.15	0.046	7.2 × 10^−5^	[16]
rs4728142	7	128,361,203	*TNP03-IRF5*	0.497	0.449	1.23	3.2 × 10^−3^	1.23	0.002	2.1 × 10^−5^	[6,11]
rs10488631	7	128,381,419	*TNP03-IRF5*	0.156	0.114	1.50	2.4 × 10^−5^	1.36	0.001	1.6 × 10^−7^	[6,7,11]
rs12531711	7	128,404,702	*TNP03-IRF5*	0.156	0.113	1.51	2.1 × 10^−5^	1.38	0.001	8.6 × 10^−8^	[11]
rs1378942	15	72,864,420	*CSK*	0.379	0.319	1.27	8.7 × 10^−4^	1.17	0.023	7.8 × 10^−5^	[11]

Chr chromosome; bp, base pairs; MAF, minor allele frequency; OR, odds ratio.

The strongest SNP association (Table 1) was with rs2857130 in the MHC. Testing imputed MHC alleles for association, the strongest signal was for DRB1*11:04 (Table 3). There was also a protective association with DRB1*07:01, but no other alleles or SNPs showed evidence of association after conditioning on these two. In particular, there was little evidence of association with the rare DRB1*11:03 allele, which only differs from DRB1*11:04 at one site encoding part of a hypervariable, peptide-binding region (R71E; OR 1.46, *P* =0.35), and a model with DRB1*11:03 and DRB1*11:04 dosage combined does not fit as well as a model with DRB1*11:04 dosage alone.

**Table 3:** **Association testing results for MHC alleles** DRB1*11:04 **and** DRB1*07:01

	**Results for** DRB1*11:04			**Results for** DRB1*07:01		
**Group**	**Mean allele dosage**	**OR (95% CI)**	***P*** **value**	**Mean allele dosage**	**OR (95% CI)**	***P*** **value**
Controls (n =4458)	0.037	1 (ref)		0.287	1 (ref)	
Cases (n =486)	0.105	3.07 (2.00 − 4.71)	2.8 × 10^−7^	0.167	0.58 (0.46 − 0.74)	1.4 × 10^−5^
ACA-positive (44% of cases)	0.080	2.46 (1.28 − 4.73)		0.105	0.34 (0.22 − 0.53)	
ACA-negative (56% of cases)	0.118	3.62 (2.13 − 6.16)	0.19^*^	0.213	0.79 (0.59 − 1.07)	0.002^*^
Scl70-positive (16% of cases)	0.174	8.22 (3.74 − 18.1)		0.294	1.28 (0.79 − 2.06)	
Scl70-negative (84% of cases)	0.088	2.47 (1.52 − 4.02)	0.021^*^	0.145	0.49 (0.37 − 0.65)	0.0012^*^
ACA & Scl70-negative (40% of cases)	0.095	2.61 (1.36 − 5.00)		0.144	0.66 (0.46 − 0.96)	

^*^These *P* values are tests of heterogeneity, comparing allele dosages in cases positive and negative for the two antibodies. Mean dosage = mean expected number of copies of alleles carried by individuals in group. OR (odds ratio) = increase in odds of being a case for each 1-unit increase in allele dosage. MHC, major histocompatibility complex; CI, confidence interval; ACA, anti-centromere antibody.

Consistent with previous findings, DRB1*11:04 is a particularly strong risk factor for Scl70-positive SSc [28] (Table 3). However, compared to controls the frequency of this allele is also elevated in ACA-positive cases and in cases negative for both antibodies. The protective effect of DRB1*07:01 is strongest against ACA-positive disease, and there was no evidence that this allele protects against anti-Scl70 antibody-positive disease.

The most significant *STAT4* locus association was at the SNP rs13426947 (Table 1). This was one of 15 associated intronic SNPs in *STAT4* (*P* <3 × 10^−4^), all correlated with rs13426947 (*r*^2^ > 0.55, MAF 0.19 to 0.26). These included the top-ranked SNP at this locus from a previous SSc GWAS (rs3821236, *P* = 2.6 × 10^−5^, *r*^2^ = 0.97 with rs13426947) [9]. No SNPs in this region were significantly associated with SSc (*P* <0.01) after conditioning on rs13426947. rs13426947 also showed association in the replication set (*P* = 5.4 × 10^−3^), and in the combined analysis (*P* = 6.1 × 10^−7^).

At the *IRF5/TNPO3* locus, there was a group of 23 associated SNPs (*P* <5 × 10^−5^) highly correlated with the top-ranked SNP rs34381587 (*r*^2^ > 0.90, MAF 0.11 to 0.13), including the top-ranked SNP reported previously (rs10488631, *P* = 2.4 × 10^−5^, *r*^2^ = 0.998 with rs34381587) [9]. There was some evidence of association with a rare SNP between the two genes (128,379,270 base pairs, MAF 0.028, OR 1.83, *P* = 6.0 × 10^−4^; *P* = 0.05 after correction for the 83 independent tests). rs34381587 also showed association in the replication set (*P* = 8.7 × 10^−4^), and near genome-wide significance in the combined analysis (*P* = 5.2 × 10^−8^).

**Figure 2:**
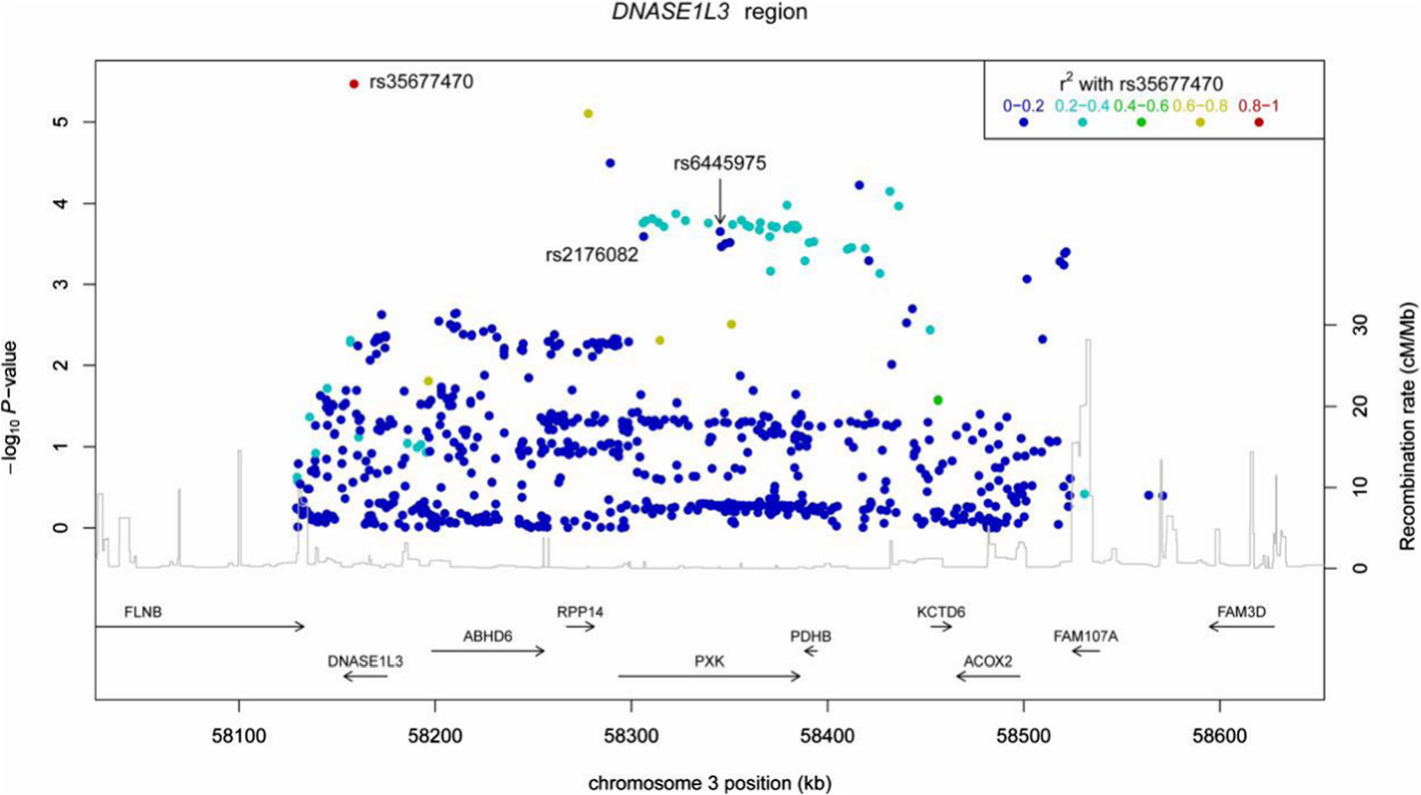
**Locus zoom plot of chromosome 3p14 region associated with scleroderma.**

Association was observed and replicated at chromosome 3p14, spanning *DNASE1L3* to *AXOX2* (including *PXK*; Figure 2), a region that has previously been associated with both SLE [29] and SSc [7]. While the peak association with SLE was originally identified at an intronic SNP rs6445975 in *PXK* (a PX domain containing serine/threonine kinase), the strongest association with SSc on the Immunochip was at a missense SNP rs35677470 (R206C) in *DNASE1L3* (deoxyribonuclease I-like 3); 187 kb distal of rs6445975. Linkage disequilibrium between the two SNPs is modest (*r*^2^ = 0.13), and there is weak evidence of a secondary association with rs6445975 in our data after conditioning on rs35677470 (OR = 1.19, *P* = 0.03). No other associations at this locus are significant after correction for multiple testing. The association with rs35677470 is confined to ACA-positive cases (estimated OR 2.36, *P* = 2.3 × 10^−10^), with no association in ACA-negative cases (*P* = 0.76). rs35677470 also showed association in the replication set (*P* =0.027), and in the combined analysis (*P* = 1.2 × 10^−6^). As in the Immunochip analysis, in the replication set association was much stronger in the ACA-positive group (*P* = 3.0 × 10^−4^), and overall (*P* = 8.71 × 10^−13^). Association was particularly significant in limited scleroderma (*P* = 3.36 × 10^−9^ in overall dataset) compared with diffuse scleroderma (*P* = 0.57), consistent with the association of ACA antibody status with limited disease. The non-synonymous *DNASE1L3* variant is predicted to be deleterious to the protein product using *in silico* functional prediction tools including both SIFT [30] and PolyPhen [31]. Apart from the *DNASE1L3* locus, no other associations showed evidence of heterogeneity by antibody status, or between limited and diffuse SSc.

A novel intergenic SNP in *VCAM1* achieved suggestive association with SSc overall in Immunochip cases (OR = 3.31, *P* = 1.8 × 10^−6^). Association was seen with both limited (*P* = 1.8 × 10^−4^), and diffuse disease (8.9 × 10^−5^). The findings for overall and limited disease were supported in the replication dataset (*P* = 0.031 and 3.1 × 10^−3^ respectively) but not with diffuse disease (*P* = 0.7). A combined meta-analysis gave *P* values of 1.9 × 10^−7^ for all cases, 1.9 × 10^−6^ for limited and 4.3 × 10^−4^ for diffuse disease.

## Discussion

The major novel finding of this study is the significant association of a functional SNP (rs35677470) in *DNASE1L3* with ACA-positive SSc (*P* = 2.3 × 10^−10^). This locus has been previously reported in a SLE GWAS [29] and meta-analysis [32], however the peak associations were reported for SNPs in the nearby gene *PXK*. While the *PXK* and *DNASE1L3* associations may be independent, no SNPs on the Illumina HumanHap300 chip used in the SLE GWAS are good tags for the missense SNP in *DNASE1L3* (maximum r^2^ of 0.21)*.* More recently, a functional variant in *DNASE1L3* was implicated in a familial form of SLE [33], and a pan-meta-analysis of SLE and SSc also confirmed the locus near *PXK*, but in particular for ACA-positive SSc (rs2176082 [7]; *P* = 1.4 × 10^−4^ in our data) strengthening the evidence that this locus plays a role in both diseases. These findings were independently identified in a study published whilst the current manuscript was in review [34].

The associated missense *DNASE1L3* variant in our data, (rs35677470 encoding R206C) affects a highly conserved residue and there is very strong evidence that this results in loss of function of the protein [35]. *DNASE1L3* encodes a member of the DNase family and functions as an endonuclease capable of cleaving DNA, mediating the breakdown of DNA during apoptosis. Al-Mayouf *et al.* [33] hypothesised that, in the context of SLE, dysfunction of this gene may lead to impaired DNA breakdown and clearance from apoptotic cells, resulting in the formation of self-directed DNA-specific antibodies and immune complexes. Since the same kinds of DNA-driven immune complexes (such as anti-nuclear and ACA antibodies) are also characteristic of SSc, this hypothesis is also applicable.

Suggestive association was also observed between a novel SNP in *VCAM1* and overall scleroderma and in limited disease cases, both of which were also associated in the replication dataset. *VCAM1* has not previously been reported to be associated with scleroderma or SLE. VCAM-1 is a member of the Ig superfamily and encodes a cell surface sialoglycoprotein expressed by cytokine-activated endothelium. It mediates leukocyte-endothelial cell adhesion and signal transduction. VCAM-1 levels have previously been shown to be elevated in early, inflammatory-phase scleroderma [36] and in limited scleroderma [37].

There were no stand-out functional variants at the *STAT4* and *IRF5/TNPO3* loci. These associations included many highly correlated SNPs, indicating that larger sample sizes and/or functional studies will be needed to understand and dissect these associations. It would, however, be prudent to investigate any functional variation at these loci identified in related autoimmune diseases, particularly SLE. The other novel associations are merely suggestive and require confirmation in additional datasets.

## Conclusions

There is a significant association of a functional SNP in *DNASE1L3* with anti-centromere antibody-positive SSc, previously reported in SLE. There is strong evidence for a loss of function of the protein. A novel association was also observed and replicated with an intergenic SNP in *VCAM1*.

This study serves to highlight that, even with a small but well-characterised disease cohort, significant associations can be obtained by tools such as the Immunochip, which are targeted towards analysis of disease-relevant and occasionally functional variation.

## Additional file

Additional file 1: Figure S1 Q-Q plot considering reading and learning disability, psychosis and schizophrenia SNPs, excluding the MHC.

## Abbreviations

ACA: anti-centromere antibody
bp: base pair
Chr: chromosome
CI: confidence interval
GWAS: genome-wide association study
MAF: minor allele frequency
MHC: major histocompatibility complex
OR: odds ratio
SLE: systemic lupus erythematosus
SNP: single nucleotide polymorphism
SSc: systemic sclerosis
