# Supplementary material for: An Immunochip-based interrogation of scleroderma susceptibility variants identifies a novel association at DNASE1L3
Source: Arthritis Res Ther. 2014 Oct 21;16(5):438. doi: 10.1186/s13075-014-0438-8 (PMC4230517; doi:10.1186/s13075-014-0438-8)

Supplementary Figure 1: QQ plot considering Reading and Learning disability, psychosis and schizophrenia SNPs, excluding the MHC.
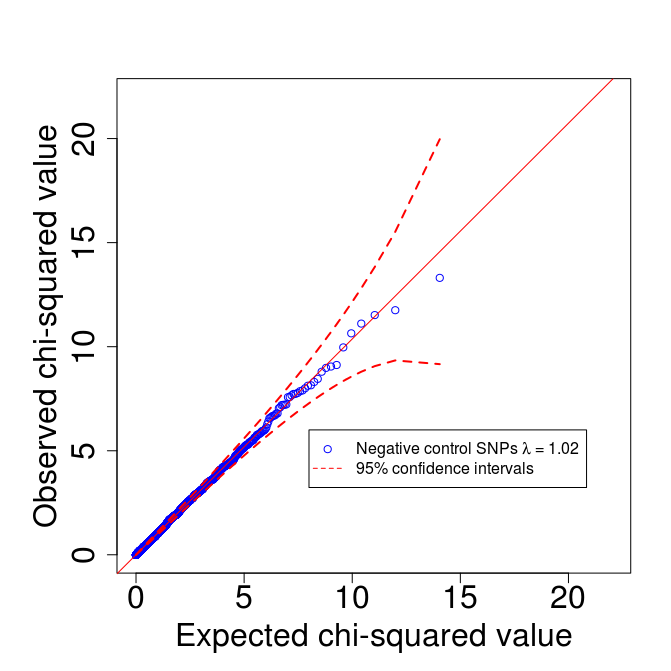

Supplement: Additional file 1: Figure S1 — Q-Q plot considering reading and learning disability, psychosis and schizophrenia SNPs, excluding the MHC. [file 13075_2014_438_MOESM1_ESM.docx]
